# Risk Factors for Premature Aging in Childhood Cancer Survivors

**DOI:** 10.34763/devperiodmed.20192302.97103

**Published:** 2019-07-08

**Authors:** Maryna Krawczuk-Rybak, Eryk Latoch

**Affiliations:** 1 Department of Pediatric Oncology and Hematology, Medical University of Białystok, Białystok, Poland

**Keywords:** aging, long-term cancer survivors, survivors of childhood cancer, treatment outcome

## Abstract

Over the last decades, the overall survival rate for childhood cancer has increased from 20% to 80%, which is the result of advances in treatment Nevertheless, most data from the international registers of childhood cancer survivors (CCS) stress that this population of patients is at high risk for late sequelae and their biologica/ aging starts earlier in life. Anticancer therapy (chemotherapy, radiotherapy, surgery, immunotherapy) affects the intracellular processes leading to the chronic deterioration of organ function and premature senescence. The present review focuses on the late effects of anticancer treatment on various human organs that may lead to premature aging.

## Introduction

Due to new anticancer therapies, the survival rates for childhood cancer have dramatically improved. Over the last 4 decades, the overall figure of such patients has increased from 20% to 80%. It is estimated that one in every 640 people had cancer in childhood and over 30 million long-term cancer survivors exist worldwide [1]. Nevertheless, one recent study covering a large number of subjects showed that more than 60% of the survivors suffered from at least one chronic condition and 30% developed severe or life-threatening sequelae [2]. Most data from large European and American registers of childhood cancer survivors (CCS) emphasize that this population is at high risk of late sequelae, and hence, their biological aging starts earlier in life.

Aging is a natura! process dependent on many intrinsic and extrinsic factors. Unfortunately, anticancer treatment (chemo-, radio-, and probably immunotherapy) influences on many intracellular processes leading to accelerated aging. A elear discrepancy between biological and chronological age in cancer survivors has been observed. For instance, the estimated life expectancy after bone marrow transplantation is 30% lower than in the generał population. The data from the Childhood Cancer Survivor Study indicated a higher incidence of an array of chronic diseases by the time these patients reach the age of 45 years in comparison with their healthy siblings: coronary heart disease (5.3% vs. 0.9%, respectively), heart failure (4.8% vs. 0.3%), arterial hypertension (up to 14.9% in CCS), stroke (77 per 100,000 vs. 9.3 per 100,000 a year), second cancers (a 5.1-5.7-fold risk increase). Cataract was observed in 40% of the survivors after head radiotherapy, osteopenia/osteoporosis in 7.6%, and metabolic syndrome in 30% at the age of 32 years. They more often develop diabetes mellitus (a 1.8-fold difference) and are at an increased risk for hypogonadism [3, 4].

## Factors leading to premature aging in childhood cancer survivors

Due to the progress in diagnosis and the development of new treatment strategies, growing numbers of CCS have been observed. Effective therapies result in an increased population of patients freed from their primary diseases but predisposed to others brought on by anticancer treatment. Moreover, their quality of life (and health span) is lower, generalhealth status (and lifespan) deteriorates compared to same-age individuals without cancer. The survivors are predisposed to early onset of chronic diseases characteristic for the elderly population. Premature aging results from the damage of normal tissues, alterations in the repair processes in normal cells in relation to primary cancer, and exposure to chemo- and/or radiotherapy. Specifically, anticancer treatment can induce premature senescence of different tissues due to the intracellular changes in deoxyribonucleic acid (DNA) structure, gene expression, mutations mediated by transcription regulator cascades, elevated expression of some proteins (such as pl6^INK4a^), or increased exposure to metabolite damage, e. g. by reactive oxygen species. When combined, these alterations lead to the so-called senescence-associated secretory phenotype, (SASP), and exert direct influence on the development of chronic diseases, such as atherosclerosis, pulmonary fibrosis, osteoporosis, cataract and diabetes [3, 5, 6].

Chemotherapy with doxorubicin or cisplatin damages mitochondrial DNA leading to neural and muscular dysfunction and a decreased ability of regeneration. Some cytostatic medications, such as cyclophosphamide and mitomycin C, as well as the modality of irradiation, both lead to altered DNA methylation. The latter process is associated with dysregulation of gene expression and cellular senescence.

Radiation and chemotherapeutic agents also induce accelerated telomere shortening, which is characteristic for many aging populations and accompanies increased expression of proinflammatory cytokines, including interleukin-6, tumor necrosis factor-a, and interleukin-1β. The length of telomeres, an important genomic DNA attribute, is responsible for the cells replication capacities and is related to DNA protection against damage. A shorter leukocyte telomere length in leukemic patients has been established as one of the risk factors for the development of second cancer [5]. The shortening of telomeres was found to be dose-dependent, along with the direct action of such cytostatic agents as cisplatin.

## Organ failure

The possibility of developing chronic health conditions in the future, e.g. the risk for second cancers, pulmonary and cardiovascular diseases, endocrinopathies, obesity, hypertension, iron overload, and bone, kidney and liver disease, is approximately 3-fold higher in CCS than in their non-affected siblings [7, 8, 9]. The survivors often develop more than one chronic health problem. In the Polish population, we observed the normal function of all organs without any detectable clinical impairment only in 11.75% of the survivors, and out of these 13.57% had at least one symptom or complaint suggesting organ dysfunction, while 15.56% presented with dysfunction of two organs, 14.14% with dysfunction of three organs, whereas in as many as 44.97% four or more organs were affected [10].

## Cardiovascular diseases

In reports from the St Jude Lifetime Cohort Study, deteriorated cardiac function was found in circa 56% of the survivors exposed to cardiotoxic therapies [3, 11]. Radiotherapy and some chemotherapeutic agents are known to be risk factors predisposing to the premature development of vascular diseases. Mediastinum irradiation for Hodgkins lymphoma (HL) and head and neck irradiation may include the areas of the carotid artery leading to its injury and predisposing to carotid arterial disease. Then, the first signs of increased intima media thickness can appear as early as one year after the end of treatment, a finding not seen in the healthy population of a similar age. Radiotherapy leads to morphological changes in the heart's atrioventricular valves with such clinical presentations as tricuspid and/or bicuspid valve regurgitations, valvular strictures, ischemic lesions of the arterial wall, injury to *vasa vasorum*, as well as the loss of elasticity, resultant fibrosis, predisposition to chronic inflammation, and impaired function of endothelial cells. Cranial irradiation and total body irradiation (TBI) can affect the functioning of the hypothalamic-pituitary axis, with a particularly harmful impact on the production of the growth hormone (GH). Secondary to abnormalities of this axis and to GH deficiency are lipid disturbances, obesity, insulin resistance, and arterial hypertension. All the above factors predispose to vascular damage of varied degree and premature atherosclerosis [12]. Radiotherapy directed at the head or the gonads and systemic chemotherapy with alkylating agents can both induce hypogonadism and, secondary to such treatments, result in the increased risk for cardiovascular disease [13]. The treatment with anthracyclines generates oxidative stress, which provokes alterations in the morphology of the cardiomyocytes. The general consequences are cardiac damage and a higher incidence of asymptomatic or symptomatic cardiac dysfunction. The changes observed in echocardiography include abnormal left ventricular systolic function and decreased ejection and shortening fractions. Over time, patients treated for HL and leukemias present with an increased risk for ischemic heart disease and myocardial infraction [14].

## Lung diseases

Abnormal pulmonary function was observed in 65.5% of the survivors who had been exposed to thoracotomy and/or pulmonary toxic cancer treatment. Radiotherapy and chemotherapy used in the treatment of childhood cancers can cause chronic lung damage and involve the highest mortality risk (standard mortality ratio: 8.8), which gradually increases with time elapsed from this treatment. It was observed that in patients diagnosed with Hodgkin’s Lymphoma (HL) whose pulmonary function tests were normal at the end of their therapy, the development of lung abnormalities occurred from 1 to 7 years later [6, 15].

The lungs are one of the most sensitive organs to radiotherapy [13, 16]. Thorax/lungs and craniospinal irradiation during childhood lead to abnormal development of the thorax, diffuse lung fibrosis, reduced lung volume, and changes in the lung parenchyma. Abnormal respiratory tests and signs of restrictive lung disease are observed after the treatment of HL (mantle irradiation), Wilms tumor (irradiation of lung metastases), and acute lymphoblastic leukemia (cranial irradiation). Overall, such irradiation may result in reduced exercise capacity. Exposure to some chemotherapeutic agents, such as bleomycin, carmustine, lomustine, busulfan, cyclophosphamide, vincristine, doxorubicin, and methotrexate with bleomycin, can all induce the development of bronchiolitis obliterans organizing pneumonia, eosinophilic hypersensitivity, lung fibrosis, and interstitial pneumonia, which finally progress to fibrosis. Megachemotherapy and TBI applied before bone marrow transplantation, thoracic surgery, graft-versus-host disease, and severe lung infections lead to lung injury and provoke restrictive ventilatory deficits with the progressive deterioration of pulmonary function, and are the major contributing factor to post-transplantation morbidity and mortality [17].

## Endocrinopathies

Symptoms of hormonal dysfunction may appear in the course of anticancer treatment, but often become manifest many years following therapy. As far as the prevalence of abnormal hormonal function is concerned, the hypothalamus-pituitary axis abnormalities or thyroid or gonadal dysfunction were observed in as many as 61% of CCS. Altered hypothalamic-pituitary function and growth hormone deficiency are seen following surgery performed within or near the hypothalamic-hypophyseal region and after cranial irradiation with doses > 18 Gy [18, 19]. GH influences not only the individual's height and physical maturation but also reduces adipose tissue content, increases muscle mass, improves glucose homeostasis, and positively affects general well-being and cognitive function. In healthy aging men, lowered GH secretion is referred to as “somatopause” and is closely related to changes in the body composition, decreased muscle mass, adiposity, metabolic and functional disorders, as well as neurocognitive deficits. GH/insulin-like growth factor-1 deficiency is associated with a higher risk for coronary artery disease and ischemic stroke. In older people, low GH secretion leads to sarcopenia and frailty and is associated with impairments in short-term memory and executive function [20, 21].

Cranial irradiation with higher doses (>30 Gy) can provoke the adrenocorticotrophic hormone, thyroid-stimulating hormone, and luteinizing hormone/follicle-stimulating hormone deficiencies with subsequent hypothyroidism, insufficient cortisol secretion, and inadequate peripheral concentrations of gonadal sex hormones (estrogens and progesterone - in women; testosterone - in men), respectively [18]. The most common endocrinopathy after childhood cancer treatment is primary thyroid dysfunction, occurring in the form of subclinical or overt hypothyroidism. Unfortunately, it is often observed after the treatment for HL (by neck/ mediastinum irradiation), craniospinal irradiation, treatment for neuroblastoma with ^131^I-metaiodobenzylguanidine, or use of kinase inhibitors, such as sorafenib, sunitinib, and imatinib. Overt (but not subclinical) hypothyroidism can be associated with the impairment of physical and cognitive function, depression, and metabolic disturbances and, over time, can provoke a higher incidence of coronary heart disease, heart failure, and even cardiovascular mortality [6, 22].

Irradiation of the minor pelvis or gonads and the treatment with alkylating agents (such as cyclophosphamide or busulfan) can seriously decrease the reproductive capacities of the patient, leading to azoospermia/ oligospermia, Leydig cell damage and lower testosterone secretion in men, and premature ovarian insufficiency, (POI), with estrogen deficits in women. Sertoli cells are more sensitive to irradiation (doses >1.0 Gy already make them vulnerable), whereas deterioration of Leydig cells is observed after 10-fold higher doses [23, 24]. Hypoestrogenism in women impairs a particularly wide array of target tissues and organs. For instance, for the bones it signifies their low mineral density, osteopenia, and osteoporosis. For lipid metabolism, it results in abnormal lipid profile with elevated triglycerides and LDL- cholesterol (or low-density lipoprotein cholesterol) levels and low HDL-cholesterol (high-density lipoprotein cholesterol) levels, thus promoting atherosclerosis as the outcome. For the cardiovascular system, besides the above-mentioned predisposition to the stiffening of the blood vessels, arterial hypertension and endothelial dysfunction are implied. Hypoestrogenism also stands behind autonomic nervous system dysfunction and a higher incidence of the metabolic syndrome due to the disturbed actions of insulin. It evokes urogenital atrophy and adversely affects the womans psychological and sexual health.

In girls treated during their late childhood and adolescence, anticancer therapy with either alkylating agents, or pelvic irradiation induces a substantial decrease of the number of oogonia/oocytes in the ovaries. This translates into diminished fertility capacities, shortened “fertility window” (timespan for possible conception), and premature menopause [25]. Importantly, when left untreated, hypogonadism in CCS affects not only fertility and sexual function but may also result in adverse effects on the cardiovascular system, bones (premature osteoporosis), increased risk for the metabolic syndrome and diabetes type 2 and, in consequence, diminished physiological reserves, frailty, and depression [26].

## Metabolic syndrome

Hypothalamic tumors, cranial or abdomen irradiation, glucocorticoids used in the treatment for acute lymphoblastic leukemia, lymphomas, brain tumors or low physical activity, can all trigger and then maintain persistent overweight and obesity. Abnormal body fat distribution (a higher accumulation in the abdominal area, even in nonobese survivors), decreased proportion of lean body mass, abnormal glucose metabolism, hyperinsulinemia, insulin resistance or overt type 2 diabetes mellitus are characteristic features of the metabolic syndrome. In cancer survivors, a spectrum of dyslipidemia symptoms can be observed, including hypertriglyceridemia, hypercholesterolemia, and high LDL- and low HDL-cholesterol concentrations. Proinflammatory and prothrombotic cytokines produced in the adipose tissue contribute to the development of atherosclerotic cardiovascular changes [27].

## The nervous system

A high prevalence of neurosensory, neurocognitive, and neurologic deficits is observed especially after treatments for brain tumors. As a result, survivors experience attention and memory deficits, learning difficulties in mathematics, problems with using receptive and expressive language, and overall decreased intelligence quotient scores. Young age at the time of treatment, high doses of irradiation, systemic and intrathecal chemotherapy with cytarabine and/or methotrexate can all induce severe progressive neurocognitive deficits [28]. Head irradiation >30 Gy and platinum agents (cisplatin, carboplatin) provoke adverse neurosensory outcomes, such as hearing loss and tinnitus [29]. Irradiation of eye areas and glucocorticoids and/ or busulfan use can lead to the development of cataracts [30]. Peripheral neuropathy may occur following the treatment with vinca alkaloids (vincristine, vinblastine) or platinum [31]. Over the years, neuromuscular impairment has been seen not only in survivors of brain tumors but also after the treatment of leukemias, lymphomas, and solid tumors [32].

## Second cancers

The risk of developing second cancers is 6-fold higher in CCS than in the general population. The type of primary cancer, its treatment modalities, and genetic predisposition are the most important risk factors. Acute myeloid leukemia and myelodysplastic syndrome are observed after chemotherapy with alkylating agents or topoisomerase II inhibitors. Radiotherapy is associated with the risk of developing skin cancers (in the irradiated areas), breast, lung, thyroid cancers, and brain, bladder and bone tumors. The risk is increased when the child was treated at a young age or when higher doses of radiation were applied. Children with primary neoplasms, such as HL, brain tumors, and acute lymphoblastic leukemia are particularly predisposed to second cancers [33]. An increased risk for multiple second cancers has been observed in families with Li-Fraumeni syndrome, where a germline mutation in suppressor genes is found. Patients with a genetic form of retinoblastoma demonstrate an increased risk for osteosarcoma. Moreover, the polymorphism of genes responsible for drug metabolism and transport or for DNA repair can also translate into the susceptibility to develop second cancers.

## Frailty

CCS are predisposed to the increased occurrence of symptoms of chronic fatigue and frailty. The frailty phenotype consists of: lean muscle mass, exhaustion, slowness, weakness, and low energy expenditure. The prevalence of the frailty phenotype (when at least 3 of the above-mentioned components are present) was observed in 2.7% male and 13.1% female CCS. Prefrailty (2 components are present) was found in 12.9% of males and in 31.5% of females. A high incidence of frailty and prefrailty is observed in survivors of brain tumors, soft tissue sarcomas, leukemias, and lymphomas. Radiotherapy, low (<18.5 kg/m2) or high (>30.0 kg/m2) body mass index, and smoking are the additional factors leading to frailty. The incidence of frailty symptoms increases with age and women are more predisposed to this phenotype. Similarly to the adult aging population, frailty in CCS is a risk factor for the development of other chronic health conditions and an independent predictor for mortality [34, 35].

Low physical activity, lean body mass, and decreased muscular strength are observed in the majority of patients treated for cancer during childhood and adolescence, however, these are the two key periods for appropriate mental and physical development. Unfortunately, in many CCS, recovery achieving general well-being is impossible due to recurrent infections, physical disability, appearance of other late effects and chronic diseases, as well as accelerated aging.

## The skeletal system

Anticancer treatment can produce latent or subclinical changes in the bones that may become clinically significant with aging. This is the result of the therapies that had been administered, exposure to steroids, and cancer itself. Among chemotherapeutic drugs, the best-established negative effect on bone mineral density (BMD) is exerted by corticosteroids and methotrexate. Bone deficits caused by alkylating drugs may appear secondary to gonadal impairment and the risk is related to cumulative dosages used. Alterations in bone metabolism may interfere with the timeframe of achieving peak bone mass, which is normally attained during the third decade of life [36]. In turn, bone mass in the elderly depends on the peak bone mass acquisition. All factors with adverse effects on the bones associated with cancer treatment in CCS will cause premature senescence and will lead to the loss of bone mass in the elderly. One study conducted on a large cohort of CCS and with a median follow-up over an impressive period of 17 years identified osteopenia in as many as 45% of the patients [37].

The most prevalent disorder affecting the skeletal system in adult CCS is osteoporosis. It may appear prematurely and manifest in severe fractures (such as fractures of the femur) at an early age. Both genders are at an equal risk for reduced BMD, however, Caucasians more so than African Americans. Patients who underwent peripheral blood stem cell transplantation are at a particularly increased risk due to the more harmful treatments applied (mega-dose chemotherapy, TBI, and high-dose alkylating agents). In these subjects, a 10% loss of BMD significantly increases the risk for subsequent fractures [38].

Besides cytostatic drugs, radiotherapy has a detrimental effect on bone health and accelerates the aging processes. In most cases, impaired mineralization in the bones is a result of earlier irradiation of the neuroendocrine axis where doses > 18 Gy can evoke GH deficiency, and doses >40 Gy induce gonadotropin deficiency. Importantly, thyroid or gonadal irradiation can trigger similar effects. Local doses >40 Gy applied directly to the bone may cause radiation-induced fractures without systemically reduced BMD.

Avascular necrosis (AVN) is nowadays a well-known complication of childhood acute lymphoblastic leukemia. AVN is an extremely rare condition in the general population, but it was found that CCS had a 20-year cumulative incidence of 0.43% and a relapse rate of 6.2 (95% CI, 2.3-17.2) compared to their siblings. Patients who were above 16 years of age at diagnosis were 6 times more likely to develop AVN than those who started therapy during their first 4 years of life [16]. The studies that were conducted show that in most cases AVN develops many years after treatment, which has a negative impact on the skeletal system in adults.

Other clinically significant problems of the skeletal system, such as scoliosis or bone and cartilage hypoplasia or atrophy, can all contribute to premature aging as well.

## Kidney diseases

Survivors of malignancy in childhood experience early and late renal adverse effects of anticancer treatment, leading to lifelong changes in their health status. Nephrotoxicity is a well-known side effect of multimodal treatment including cytostatic drugs (cisplatin, carboplatin, cyclophosphamide, ifosfamide), immunotherapy, supportive treatment (aminoglycoside antibiotics, amphotericin, furosemide), radiation therapy to the abdomen, and surgery [39]. A number of distinct clinical pictures can develop, including acute kidney injury (observed more often during aggressive treatment or being associated with tumor lysis syndrome), chronic kidney disease, proteinuria, arterial hypertension, and hyperfiltration. CCS have a 9-fold higher risk of developing renal failure in comparison with their siblings [2, 40].

Ifosfamide intake can lead to the development of glomerulopathy and renal Fanconi’s syndrome (glycosuria, phosphaturia, and amino aciduria) and, subsequently, to abnormal growth and bone diseases [6]. According to one of the largest studies assessing glomerular function, glomerular filtration rate in survivors who had received nephrotoxic treatments was lower compared with those who had not, and the increased probability of glomerular dysfunction extended to up to 35 years after treatment. Moreover, glomerular filtration rate continued to decrease with time [41]. Currently, chronic kidney disease is the most prevalent health problem in the elderly worldwide. Available data derived from CCS show that their kidneys may have been damaged earlier in life. Their chronic kidney disease can progress to end-stage kidney failure, when patients require hemodialysis and transplantation.

## Conclusions

As discussed above, CCS are a growing high-risk population that requires dedicated healthcare throughout their whole lifespan. There is a need for a better understanding of late effects of cancer and its treatment on the processes of aging. The education of patients and survivors and their awareness of the occurrence of late effects which can seriously impact their future life is very important. Prophylaxis (by means of a proper diet, physical activity, self-control, cessation of tobacco smoking, etc.), regular health checkups in line with guideline recommendations, as well as the awareness of healthcare providers, especially family doctors, can minimize these late sequelae. It is necessary to create new healthcare delivery system designs to customize each individual CCS's needs depending on their medical history. Such solutions will both improve the general health status and quality of life of CCS and reduce the costs of treating many of their diseases.

## Abbreviations

AVN: avascular necrosis
CCS: childhood cancer survivors
BMD: bone mineral density
DNA: deoxyribonucleic acid
GH: growth hormone
HDL: cholesterol – high-density lipoprotein cholesterol
HL: Hodgkin’s lymphoma
LDL: cholesterol – low-density lipoprotein cholesterol
TBI: total body irradiation
